# Estimating the energy expenditure of free‐ranging polar bears using tri‐axial accelerometers: A validation with doubly labeled water

**DOI:** 10.1002/ece3.5053

**Published:** 2019-03-19

**Authors:** Anthony M. Pagano, Terrie M. Williams

**Affiliations:** ^1^ Alaska Science Center U.S. Geological Survey Anchorage Alaska; ^2^ Department of Ecology & Evolutionary Biology University of California, Santa Cruz Santa Cruz California; ^3^ Present address: Institute for Conservation Research San Diego Zoo Global San Diego California

**Keywords:** dynamic body acceleration, energetics, field metabolic rate, oxygen consumption, polar bear, *Ursus maritimus*

## Abstract

Measures of energy expenditure can be used to inform animal conservation and management, but methods for measuring the energy expenditure of free‐ranging animals have a variety of limitations. Advancements in biologging technologies have enabled the use of dynamic body acceleration derived from accelerometers as a proxy for energy expenditure. Although dynamic body acceleration has been shown to strongly correlate with oxygen consumption in captive animals, it has been validated in only a few studies on free‐ranging animals. Here, we use relationships between oxygen consumption and overall dynamic body acceleration in resting and walking polar bears *Ursus maritimus* and published values for the costs of swimming in polar bears to estimate the total energy expenditure of 6 free‐ranging polar bears that were primarily using the sea ice of the Beaufort Sea. Energetic models based on accelerometry were compared to models of energy expenditure on the same individuals derived from doubly labeled water methods. Accelerometer‐based estimates of energy expenditure on average predicted total energy expenditure to be 30% less than estimates derived from doubly labeled water. Nevertheless, accelerometer‐based measures of energy expenditure strongly correlated (*r* ^2^ = 0.70) with measures derived from doubly labeled water. Our findings highlight the strengths and limitations in dynamic body acceleration as a measure of total energy expenditure while also further supporting its use as a proxy for instantaneous, detailed energy expenditure in free‐ranging animals.

## 1 INTRODUCTION

Energy expenditure is fundamental to animal movement ecology and influences survival and reproductive success and, hence, population dynamics (Brown, Gillooly, Allen, Savage, & West, 2004). Specifically, measures of energy expenditure can provide insight about the mechanisms by which animals respond to environmental change on both short‐ and long‐term scales (Wikelski & Cooke, 2006). Due to the importance of energy balance in species survival, a variety of methods have been developed to measure animal energy expenditure in the field using electronic logging and tracking devices (Wilmers et al., 2015). For example, heart rate monitors (Boothby, 1915; Butler, Woakes, Boyd, & Kanatous, 1992; Krogh & Lindhard, 1917) and more recently accelerometers have been developed, which provide data that can be used as a proxy for energy expenditure (Wilson et al., 2006). Similar to heart rate monitors (Butler, Green, Boyd, & Speakman, 2004), accelerometers can be calibrated with measures of oxygen consumption as a means to quantify overall energy expenditure (Gómez Laich, Wilson, Gleiss, Shepard, & Quintana, 2011; Halsey et al., 2009; Halsey, White et al., 2011; Wilson, Quintana, & Hobson, 2012; Wilson et al., 2006).

**Figure 1:**
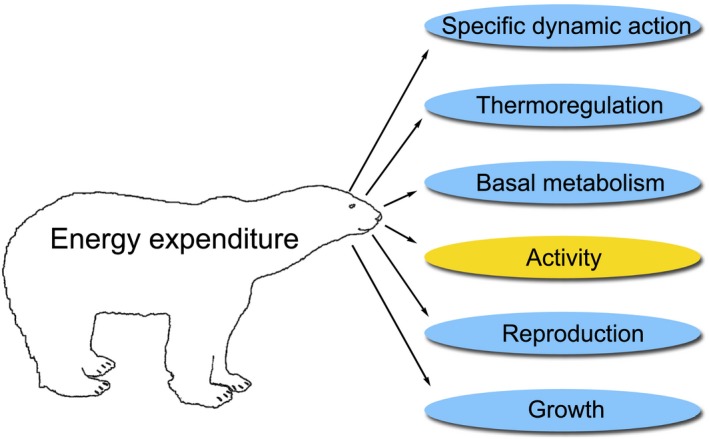
Conceptual chart showing the energetic pathways that account for an animal's overall metabolizable energy. The doubly labeled water method measures potential changes in energy expenditure across all of these pathways. Conversely, the accelerometer method only accounts for potential changes in energy expenditure that result from changes in activity; the remaining metabolic costs must be accounted for in energetic models to determine total metabolic rate or field metabolic rates over longer time periods (See Williams, Fuiman, Horning, & Davis, 2004)

The translation of accelerometer data into a measure of energy expenditure has been termed dynamic body acceleration (DBA; Wilson et al., 2006), which represents changes in velocity as a result of animal body movements (Shepard et al., 2008). The method is based on the concept that DBA provides a proxy for mechanical work performed by locomotor muscles, which should reflect changes in energy expenditure (Gleiss, Wilson, & Shepard, 2011; Wilson et al., 2006). This relies on the assumption that movement is a primary factor influencing variability in energy expenditure (Costa & Williams, 1999; Gleiss et al., 2011; Karasov, 1992; Wilson et al., 2006). Nevertheless, Green, Halsey, Wilson, and Frappell (2009) found that DBA could also predict energy expenditure when including periods of inactivity in birds. When DBA is summed across 3 dimensions, it has been termed overall dynamic body acceleration (ODBA), which provides a means to quantify body movement in all directions at the center of mass (Wilson et al., 2006). The advantages of the DBA method are that accelerometers can be externally attached, can potentially record for extended durations over multiple months, and they can provide detailed, short‐term measures of energetic costs at subsecond scales (Gleiss et al., 2011; Halsey, Shepard, & Wilson, 2011; Williams et al., 2014; Wilmers, Isbell, Suraci, & Williams, 2017; Wilson et al., 2006). This has considerable benefits in reducing the invasiveness of metabolic research relative to other methods. Given their small size and external attachment, the use of accelerometers should reduce the potential for injury (Green, Haulena et al., 2009) and reduce the potential for influencing the animal's behavior and related energy expenditure (but see Chivers, Hatch, & Elliott, 2016; Maresh et al., 2014; Vandenabeele et al., 2014; Walker & Boveng, 1995; Wilson, 2011). As a result, DBA is increasingly being used as a proxy for energy expenditure in free‐ranging animals (Bishop et al., 2015; Bryce, Wilmers, & Williams, 2017; Enstipp et al., 2016; Grémillet et al., 2018; Halsey & White, 2010; Hicks et al., 2018; Scharf, LaPoint, Wikelski, & Safi, 2016; Udyawer, Simpfendorfer, Heupel, & Clark, 2017; Wang, Smith, & Wilmers, 2017; Williams et al., 2016, 2014; Wilmers et al., 2017; Wilson et al., 2012). However, DBA may underestimate changes in energy expenditure as a result of changes in basal metabolism, thermoregulation, specific dynamic action (heat increment of feeding), reproduction, or growth (Gleiss et al., 2011; Green, Halsey et al., 2009; Halsey, Shepard et al., 2011; Figure 1).

Given the potential utility of accelerometers, there is an increasing need to assess the validity, strengths, and limitations of DBA for measuring energy expenditure in free‐ranging animals, which has only been tested in a few studies (Elliott, Le Vaillant, Kato, Speakman, & Ropert‐Coudert, 2013; Jeanniard‐du‐dot, Guinet, Arnould, Speakman, & Trites, 2017; Stothart, Elliott, Wood, Hatch, & Speakman, 2016). Currently, the doubly labeled water (DLW) method (Lifson, Gordon, & McClintock, 1955; Lifson & McClintock, 1966) is the most widely used technique for measuring total energy expenditure (otherwise known as field metabolic rate, FMR) in free‐ranging animals (Nagy, 1989; Speakman, 1997). The DLW method provides a direct average estimate of CO_2_ production over an interval of time, which can be used in place of oxygen consumption to estimate metabolic rates (Speakman, 1997). The method involves injecting a dose of water containing the isotopes ^2^H or ^3^H and ^18^O to determine the rate of CO_2_ production over the measurement period. The difference between the turnovers of the oxygen and hydrogen isotopes provides a measure of CO_2_ production because ^18^O declines from the body as both respiratory CO_2_ (efflux) and water influx while ^2^H or ^3^H decline solely as a result of water influx (Costa, 1987, 1988; Speakman, 1997). The biggest advantages of the DLW method are that it does not require calibration using captive surrogates and it can be used directly on animals in the field. However, the use of DLW entails a variety of assumptions (Butler et al., 2004; Costa, 1987; Nagy, 1980; Speakman, 1997), it typically requires capturing and sampling individuals on two occasions within a specific time frame (but see Scantlebury et al., 2014), and it only provides metabolic data during the period between equilibration at initial capture and final enrichment at recapture. Hence, the DLW method is only useful over relatively short time frames (typically over a few days) and it provides a single measure of energy expenditure making it difficult to assess the energetic costs of specific behaviors (Butler et al., 2004; Costa, 1988; Speakman, 1997). Furthermore, the purchase of ^18^O can be expensive (Speakman, 1997). Thus, the DLW method is often unsuitable or cost‐prohibitive for most field studies of large animals. Nevertheless, the DLW method can be used to validate alternative measures of energy expenditure such as the DBA method as a means of measuring the energy expenditure of free‐ranging animals.

Here, we evaluate the use of ODBA from tri‐axial accelerometers to measure the energy expenditure of a large free‐ranging carnivore, female polar bears *Ursus maritimus*, on the sea ice of the Beaufort Sea by comparing the data to simultaneous measures of energy expenditure derived using DLW (Pagano, Durner et al., 2018). The behaviors and activity rates of individual bears were identified based on a previously developed random forest model using tri‐axial accelerometer and conductivity sensor data (Pagano, Rode, Cutting et al., 2017). A relationship between oxygen consumption and ODBA was developed from measures of metabolic rate from captive, collared adult female polar bears resting and walking on a treadmill (Pagano, Carnahan et al., 2018). Energetic costs of swimming were derived from modeled estimates using internal body temperature data from free‐ranging polar bears while swimming (Griffen, 2018). Together, these data allowed us to compare accelerometer‐derived measures of energy expenditure to expenditures derived using DLW. Measures of daily energy expenditure derived from DLW were further compared to mean ODBA, mean activity rates, mean movement rates, and mean body mass to assess whether accelerometer‐derived measures of energy expenditure offer an improvement over other metrics.

## 2 MATERIALS AND METHODS

### 2.1 Captures

Data were collected from free‐ranging subadult and adult female polar bears without dependent young, on the sea ice of the Beaufort Sea as part of a previous study (Pagano, Durner et al., 2018) in April 2014, 2015, and 2016. Details of the capture methods, use of doubly labeled water, and deployment of satellite collars with tri‐axial accelerometers are described elsewhere (Pagano, Durner et al., 2018). Briefly, polar bears were located from a helicopter and immobilized with standard methods (Stirling, Spencer, & Andriashek, 1989). Following immobilization, we weighed bears using an electronic load cell suspended from an aluminum tripod. Bears that had not been previously captured were aged based on counts of cementum annuli from an extracted vestigial premolar (Calvert & Ramsay, 1998; C‐D & Associates Biological Consulting, Spruce Grove, Alberta, Canada). Procedures were approved by the Animal Care and Use Committees of the University of California, Santa Cruz and the U.S. Geological Survey, Alaska Science Center. Research was approved under U.S. Fish and Wildlife Service Marine Mammal Permit MA690038.

### 2.2 Doubly labeled water

Following immobilization, an initial blood sample was collected to serve as a baseline measure of ^18^O (oxygen‐18) and ^2^H (deuterium). The bear was then injected intravenously with a weighed dose containing 0.12–0.25 g/kg of 97% enriched ^18^O (Isoflex USA, San Francisco, CA, USA) and 0.06–0.10 g/kg of 99.9% enriched ^2^H (Isotec, Inc., Miamisburg, OH, USA or Cambridge Isotope Laboratories, Inc., Tewksbury, MA, USA) with NaCl added to make it 0.9% isotonic and sterilized using a 0.2 µ Millipore filter. On injection, the syringe was back washed with blood three times to ensure all the DLW had been injected into the bear. The bear was kept immobilized for 2 hr after the injection of DLW to allow isotope equilibration (Pagano, Rode, & Atkinson, 2017). We recaptured bears 8–11 days later to obtain a blood sample to measure final enrichment. At recapture, we weighed bears to measure changes in body mass.

Serum samples were analyzed for the concentrations of ^18^O and ^2^H (Metabolic Solutions, Inc., Nashua, NH, USA). We calculated CO_2_ production using the plateau method and Speakman's two‐pool equation (Speakman, 1997), which has been shown to be best suited for large mammals (Sparling, Thompson, Fedak, Gallon, & Speakman, 2008; Speakman, 1997; Speakman et al., 2001, but see Dalton, Rosen, & Trites, 2014). We used the mean group dilution space ratio in calculating CO_2_ production (Speakman, 1997). We converted CO_2_ production to metabolic rate using a respiratory quotient of 0.76 (26.32 kJ per liter CO_2_; Pagano, Durner et al., 2018).

### 2.3 GPS‐collars and accelerometers

We deployed GPS‐equipped video camera collars (Exeye, LLC., Bristow, VA, USA) and archival loggers (TDR10‐X‐340D, Wildlife Computers Inc., Redmond, WA, USA) on the same individuals dosed with DLW (see Pagano, Durner et al., 2018 for additional information). To evaluate differences in mean movement rates (km/hr) among bears, we used a continuous time correlated random walk (CRAWL) model (Johnson, 2016; Johnson, London, Lea, & Durban, 2008) in R (R Core Team, 2014) to predict locations on a 30 min interval based on GPS locations. We calculated minimum distance travelled between two successive predicted locations as the great‐circle distance (i.e., distance accounting for the earth's curvature) and calculated movement rate by dividing distance by the duration between predicted locations (i.e., 30 min) in SAS (version 9.3, SAS Institute Inc., Cary, NC, USA).

Archival loggers recorded tri‐axial acceleration (m/s^2^) at 16 Hz (range ± 20 m/s^2^), time‐of‐day, and wet/dry conductions at 1 Hz (via an onboard conductivity sensor) continuously from the time of deployment until recovery. Behaviors were derived from the accelerometer and conductivity data using a random forest algorithm (Breiman, 2001) in R (“RandomForest”package) as described by Pagano, Rode, Cutting et al. (2017). Briefly, we discriminated 10 behaviors from the accelerometer and conductivity data and we calculated activity to be the proportion of time a bear was not engaged in resting behaviors. To calculate DBA, we converted accelerometer measures from m/s^2^ to *g* (1 g = 9.81 m/s^2^). We used a 2 s running mean of the raw acceleration data to calculate static acceleration (gravitational acceleration) and subtracted the static acceleration from the raw acceleration data to calculate dynamic acceleration (Shepard et al., 2008; Wilson et al., 2006). We calculated ODBA as the absolute sum of dynamic acceleration across the 3 axes (Wilson et al., 2006).

### 2.4 Conversion of accelerometer measures to energy expenditure

**Figure 2:**
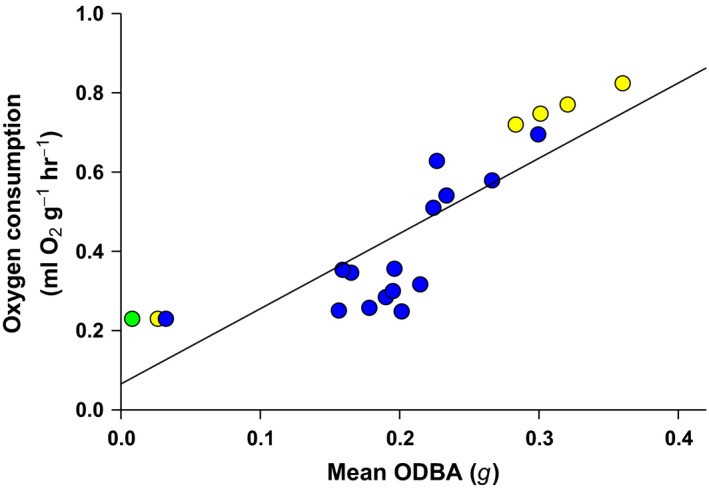
Least‐squares regression (solid line) of mass‐specific oxygen consumption and mean overall dynamic body acceleration (ODBA) from 2 adult female polar bears resting and walking on a treadmill (bears 1 and 2; yellow and blue circles, respectively) and 3 adult female polar bears resting in their enclosure (bears 1, 2, and 3; yellow, blue, and green circles, respectively). Regression statistics are provided in the main text

Energy expenditure during nonswimming behaviors was based on the relationship between ODBA and oxygen consumption (V̇O_2_) derived by Pagano, Carnahan et al. (2018). However, Pagano, Carnahan et al. (2018) found a negative intercept between ODBA and V̇O_2_ and suggested this relationship needed to be further developed. This negative intercept may have been related to head movements of the bears during resting V̇O_2_ and ODBA measurements. To correct for the potential that head movements during resting measurements biased our ODBA measures, we measured the mean ODBA of 3 captive adult female polar bears (body mass: 237, 242, and 288 kg; age: 14, 31, and 15 years) while resting and motionless in their enclosures (Pagano, Rode, Cutting et al., 2017) and assigned them the mean mass‐specific resting metabolic rate summarized by Pagano, Carnahan et al. (2018) from three studies (0.230 ml O_2_ g^−1^ hr^−1^). We incorporated these three measures with the previous measures determined by Pagano, Carnahan et al. (2018) and found V̇O_2_ (ml O_2_ g^−1^ hr^−1^) increased linearly as a function of ODBA (*g*) where: V̇O_2_ = 0.07 + 1.90 × ODBA (*r* ^2^ = 0.71, *p* < 0.001, *n = *21, Figure 2). For swimming movements, we used the mean swimming energetic cost determined by Griffen (2018) (2.75 ml O_2_ g^−1^ hr^−1^). Measures of V̇O_2_ were converted to metabolic rate using the standard conversion factor of 20.083 J/ml O_2_ (Schmidt‐Nielsen, 1997). These relationships were then applied on a behavior‐specific basis (i.e., swimming vs. nonswimming) to the archival logger data recovered from the same free‐ranging individuals that were dosed with DLW to measure their total energy expenditure (mJ/kg) in SAS. Total energy expenditure was converted to daily energy expenditure by dividing by the total number of days each animal was studied.

### 2.5 Statistical analysis

We used paired *t* tests to compare estimates of daily energy expenditure (kJ kg^−1^ day^−1^) measured by DLW and concurrent estimates derived from the accelerometer‐based relationship (i.e., the conversion of the accelerometer data into measures of energy expenditure as described above). Least‐squares linear regression was then used to evaluate the relationship between daily energy expenditure measured by DLW and estimates derived from the accelerometer‐based relationship. For each individual, we calculated mean ODBA (*g*), mean activity rate (%), and mean movement rate (km/hr) between the time of capture to the time of recapture. We used least‐squares linear regression to evaluate the relationships between daily energy expenditure (kJ kg^−1^ day^−1^) derived from DLW and (a) mean ODBA (*g*), (b) mean activity rate (%), (c) mean movement rate (km/hr), and (d) mean body mass (kg). We considered results to be significant at *p* ≤ 0.05. All analyses were conducted in R.

## 3 RESULTS

We captured four adult female polar bears in 2014, three adult and one subadult female polar bears in 2015, and two adult female polar bears in 2016. We recaptured bears 8–11 days later to obtain a blood sample to measure final enrichment and recover collars and archival loggers. In 2015, we were unable to recapture one adult female to measure her final enrichment. Additionally, three of the archival loggers deployed in 2014 failed within 12 hr of deployment. We excluded data from these four bears from analyses. Consequently, we had a sample of 6 female polar bears who provided simultaneous measures of energy expenditure derived from DLW and continuous measures of tri‐axial acceleration (Table 1). Across these 6 individuals, we collected 228,890,898 measures of acceleration across three axes of which 0.05% equaled or exceeded the maximum range of recorded acceleration (i.e., ≥ or ≤ ±20 m/s^2^).

**Table 1:** Polar bear age, duration studied, mean body mass, mean daily field metabolic rates (FMRs) derived from doubly labeled water (DLW), mean daily FMRs derived from accelerometer‐based relationships (ACC), and mean measures of overall dynamic body acceleration (ODBA)

Bear	Age (years)	Duration (days)	Mean mass (kg)	FMR_DLW_ (kJ kg^−1^ day^−1^)	FMR_ACC_ (kJ kg^−1^ day^−1^)	Mean ODBA (*g*)
1	6	9.1	184.4	363.6	282.7	0.274
2	4	9.2	189.2	290.1	243.1	0.231
3	5	10.0	171.3	274.0	182.4	0.168
4	3	10.6	133.6	214.1	155.1	0.135
5	6	8.0	209.1	402.1	269.7	0.256
6	22	8.9	205.3	337.4	199.1	0.186

**Figure 3:**
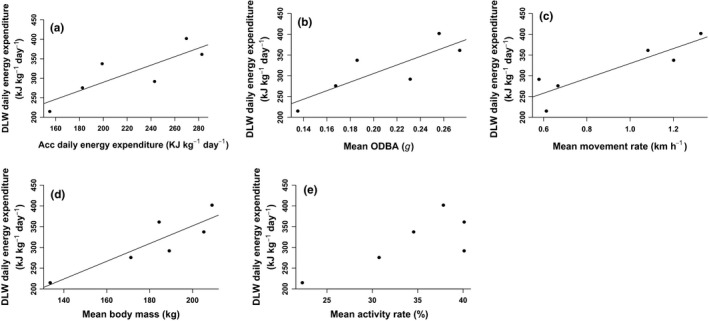
Mass‐specific energy expenditure in relation to accelerometer‐derived estimates, overall dynamic body acceleration (ODBA), movement, body mass, and activity of polar bears. (a) Least‐squares regression (solid line) of mean daily mass‐specific energy expenditure of female polar bears on the sea ice measured by doubly labeled water (DLW) in comparison with mean daily mass‐specific energy expenditure of the same individuals measured based accelerometer‐based relationships. (b) Least‐squares regression (solid line) of mean daily mass‐specific energy expenditure of female polar bears on the sea ice measured by DLW in comparison with mean ODBA. (c) Least‐squares regression (solid line) of mean daily mass‐specific energy expenditure of female polar bears on the sea ice measured by DLW in comparison to mean movement rate. (d) Least‐squares regression (solid line) of mean daily mass‐specific energy expenditure of female polar bears on the sea ice measured by DLW in comparison to mean body mass. (e) Relationship between mean daily mass‐specific energy expenditure of female polar bears on the sea ice measured by DLW in comparison with mean activity rate. Regression statistics are provided in the main text. Each point represents a single value for one bear

Daily energy expenditure derived from DLW ranged from 1.2 to 1.7 times greater than accelerometer‐derived estimates of daily energy expenditure (mean = 1.4, *SE* = 0.07, Table 1). As a result, daily energy expenditure derived from DLW differed significantly from estimates derived from accelerometers (*t*_5_ = 6.1, *p* = 0.002). Nevertheless, daily energy expenditure estimates derived from accelerometers accounted for 70% of the variation in daily energy expenditure estimates derived from DLW: DEE_DLW_ = 70.87 + 1.09 × DEE_ACC_ (*r* ^2^ = 0.70, *p* = 0.039, *n* = 6, Figure 3a). Similarly, mean ODBA accounted for 70% of the variation in daily energy expenditure estimates derived from DLW: DEE_DLW_ = 99.09 + 1031.58 × ODBA (*r* ^2^ = 0.70, *p* = 0.039, *n* = 6, Figure 3b). Mean movement rate accounted for 88% of the variation in daily energy expenditure estimates derived from DLW: DEE_DLW_ = 150.58 + 179.17 × rate (*r* ^2^ = 0.88, *p* = 0.005, *n* = 6, Figure 3c). Mean body mass accounted for 77% of the variation in daily energy expenditure estimates derived from DLW: DEE_DLW_ = −74.89 + 2.13 × mass (*r* ^2^ = 0.77, *p* = 0.02, *n* = 6, Figure 3d). Mean activity rate did not significantly explain the variation in daily energy expenditure estimates derived from DLW (*r* ^2^ = 0.60, *n* = 6, *p* = 0.069, Figure 3e).

## 4 DISCUSSION

An animal's energy expenditure is a function of many metabolic costs including basal metabolism, specific dynamic action, thermoregulation, reproduction, growth, and locomotion (Figure 1) (Costa & Williams, 1999; Nagy, 1989). For polar bears, a previous study using the same DLW measures of energy expenditure found that energy expenditure was influenced by body mass, movement rate, overall activity level, and total distance traveled (Pagano, Durner et al., 2018), suggesting that basal metabolism and movement costs were primary determinants of energy expenditure in this species. Despite this, the discrepancies we report here between energy expenditure determined via DLW and accelerometers suggest the potential influence of other costs on energy expenditure not associated with activity, such as specific dynamic action (SDA), thermoregulation, growth, and reproduction (Figure 1). Such costs are inherently incorporated within DLW estimates, but their variability could not be accounted for with the models we used in our estimates derived from accelerometers. Five of the 6 bears in this study were adults and all bears were independent females without cubs. Hence, growth and reproduction likely had minimal impacts on energy expenditure. Conversely, SDA was probably an important factor as 5 of the bears in this study either scavenged from seal and bowhead whale *Balaena mysticetus* carcasses or caught and ate adult/subadult ringed seals *Pusa hispida *(Pagano, Durner et al., 2018). In other mammals, SDA has been found to typically increase energy expenditure for 4–10 hr by 25%–50% (Costa & Kooyman, 1984; Secor, 2009). Scavenging bears likely experienced particularly high SDA costs due to the greater energetic costs in digesting protein from muscle relative to fat (Costa & Williams, 1999) and the increased energetic cost of heating frozen food to body temperature (Berteaux, 2000; Secor, 2009; Wilson & Culik, 1991). Only 1 bear fasted for the duration of the study (Pagano, Durner et al., 2018). Yet, even this bear was estimated to have 1.4 times greater energy expenditure based on DLW than estimates derived from accelerometers. Ambient temperatures during the period of this study ranged from −33.9 to 1.7°C. Best (1976) estimated that the thermoneutral zone for a 200 kg polar bear may range from −50 to 37°C while resting, which suggests that the effect of thermoregulation on the energy expenditure of the bears in this study would be expected to be minimal. Additionally, if bears were shivering or panting to thermoregulate, we would expect such responses to be detected by our accelerometers and influence our energy expenditure estimates. However, Green, Haulena et al. (2009) found accelerometers failed to detect shivering and thermoregulatory costs in domestic chickens (*Gallus gallus*). Furthermore, the bears in this study spent only 0.3% of the time swimming in the water (Pagano, Durner et al., 2018) (where thermoregulation costs may be greater). Together, this suggests that other factors beyond growth, reproduction, SDA, and thermoregulation may be contributing to the discrepancies, we found in measuring energy expenditure between the two methods.

Of particular importance is the robustness of the relationship between V̇O_2_ and ODBA to account for different terrains, gait changes, and the range of speeds of wild animals. The relationship we developed on the treadmill was limited to a maximum speed of 3 km/hr, which may have limited our ability to predict energy expenditure at greater speeds. However, in polar bears, V̇O_2_ has been shown to increase linearly at speeds up to 5.4 km/hr (Pagano, Carnahan et al., 2018) and free‐ranging polar bears, including those in this study, rarely exceed this speed (Pagano, Carnahan et al., 2018; Whiteman et al., 2015). Another factor that can influence the relationship between ODBA and V̇O_2_, and thus cost of locomotion, is uneven surfaces including moving on rough or icy surfaces, and traversing inclines and declines (Halsey, 2016; Halsey et al., 2008). Accounting for this effect requires further calibration on different substrates, and at varying inclines with determination of the slope and substrate at which animals are moving in the field (Gleiss et al., 2011). Typically, it is assumed that the relationship between V̇O_2_ and ODBA derived on a treadmill will follow similar trends in the field. This has to be approached with caution for the reasons described above as well as extraneous body movements that are known to impose greater energetic costs relative to movements on a treadmill (Halsey, 2016). For example, in humans, DBA has been shown to underestimate field measurements of V̇O_2_ (Bidder et al., 2017). We performed a post hoc test to assess whether the difference in energy expenditure derived from the two methods may be explained by differences in the activity rates among our bears, which could suggest our treadmill‐derived calibrations were driving our underestimates of energy expenditure, but we found no significant relationship (*r* ^2^ = 0.03, *p* = 0.76, *n* = 6). Hence, our results further suggest that multiple factors may lead to underestimates of field energetic costs of free‐ranging animals when using DBA techniques without appropriate calibrations (Adachi et al., 2014; Bidder et al., 2017; Dalton et al., 2014; Green, Halsey et al., 2009; Halsey, Shepard et al., 2011; Jeanniard‐du‐dot et al., 2017). Additionally, DLW measures of energy expenditure themselves are known to contain some error. Nagy (1989) and Speakman (1997) reported this error to average 4% and 3.1% in mammals, respectively. However, individual error may be as high as 44% (Butler et al., 2004; Dalton et al., 2014; Sparling et al., 2008). Hence, the discrepancies we found with energy expenditure derived from accelerometers may in part be due to errors in the DLW estimates themselves.

Although our sample size was limited, we found similar relationships with energy expenditure derived from DLW using either our estimates of energy expenditure derived from accelerometers or mean ODBA. This similarity in part likely reflects the conversion of ODBA to measures of energy expenditure based on a linear relationship for all behaviors except swimming. Additionally, the swimming frequencies of bears during the study (mean = 0.3%) was likely too low to considerably affect the relationship between energy expenditure derived from accelerometers and mean ODBA. This highlights that while conversion of ODBA to a measure of energy expenditure using a linear relationship with V̇O_2_ does convert data to units of energy (i.e., kJ kg^−1^ day^−1^), such linear conversions will not influence the ability of accelerometer‐based measures to serve as proxies for energy expenditure. Strong relationships between DBA and DLW measures of energy expenditure have similarly been shown in free‐ranging thick‐billed murres *Uria lomvia* and pelagic cormorants *Phalacrocorax pelagicus* (*r* ^2^ = 0.73 and 0.91, respectively) (Elliott et al., 2013; Stothart et al., 2016). This highlights the potential value of DBA as a proxy of energy expenditure because locomotion costs typically account for most of the variability in energy expenditures in free‐ranging animals (Costa & Williams, 1999; Gleiss et al., 2011; Karasov, 1992; Wilson et al., 2006). Nevertheless, in our study, mean movement rate and mean body mass also strongly correlated with energy expenditure. The strong correlation between energy expenditure and body mass likely reflects the important role of basal metabolism in influencing energy expenditure (Nagy, 2005; Pagano, Durner et al., 2018; Ricklefs, Konarzewski, & Daan, 1996). Movement rate would be expected to function as both a coarse proxy of activity (Ensing et al., 2014) and a measure of the intensity of such activity (i.e., speed). That movement rate strongly correlated with energy expenditure while activity rate did not suggests that locomotion costs were a more important determinant of energy expenditure than activity alone. However, like DBA, measures of movement rate and body mass have their own limitations. To measure potential changes in energy expenditure, measures of body mass require repeated sampling of individuals, which is one of the limitations in the use of DLW itself. Movement rates can be calculated from satellite telemetry location data, but for polar bears, movement rates derived by satellite telemetry can be biased by sea ice drift (Auger‐Méthé, Lewis, & Derocher, 2016; Durner et al., 2017; Mauritzen, Derocher, Pavlova, & Wiig, 2003; Platonov et al., 2014). Such effects were considered minimal in the region and month of our study (Durner et al., 2017), where previous research indicates a bias would be more prevalent in other regions and months (Durner et al., 2017). In addition to this limitation, movement rates are typically calculated from comparatively infrequent location data, thus underestimating true movement paths and rates (Bidder et al., 2015; Kramer & McLaughlin, 2001; Prichard, Yokel, Rea, Person, & Parrett, 2014; Rowcliffe, Carbone, Kays, Kranstauber, & Jansen, 2012). By comparison, measures of DBA are typically recorded at high frequencies and, hence, should better reflect an animal's true movements (Bidder et al., 2015; Wilson et al., 2013). Measures of tri‐axial acceleration can also be used to simultaneously determine animal behavior (this study; Ladds et al., 2018), providing additional insight into animal movement ecology and conservation (Cooke et al., 2014).

Although we highlight some potential limitations and cautionary measures concerning the use of DBA as a measure of energy expenditure, we recommend future research to increase its application for the wide diversity of free‐ranging animals and the habitats in which they move. Due to the expense of DLW isotopes and the logistical constraints of working with large carnivorous mammals in the Arctic, the sample size in this study was limited. Furthermore, although the bears in this study spent <1% of the time swimming, the energetic costs of swimming in polar bears has yet to be directly measured. The use of ODBA to estimate energy expenditure relies on the premise that acceleration represent movements at the animal's center of mass (Gleiss et al., 2011; Wilson et al., 2006). Similar to other studies, we used tri‐axial accelerometers mounted to collars at the neck to measure ODBA (Halsey et al., 2008, 2009; Qasem et al., 2012; Williams et al., 2014). However, it is unknown whether the relationships we derived might have been influenced by attachment on the collar and if we would have found improved relationships with the accelerometer mounted directly on the animal's trunk. Future research exploring these topics would help to improve the accuracy and reliability of measures of energy expenditure in field studies. Nevertheless, our results support the use of accelerometers in providing novel insight into the instantaneous energy expenditure of free‐ranging animals.

## CONFLICT OF INTEREST

None declared.

## AUTHORS' CONTRIBUTIONS

A.M.P. and T.M.W. designed the project. A.M.P. led data collection, conducted data processing and analysis, and writing of the manuscript. T.M.W. assisted with oxygen consumption data collection and writing of manuscript.

## Data Availability

Data reported in this paper are archived in the USGS Alaska Science Center data repository at, https://doi.org/10.5066/F7QR4W91 and https://doi.org/10.5066/F7XW4H0P.
